# White Matter Characteristics of Cognitive Impairment in Tap-Test Positive Idiopathic Normal Pressure Hydrocephalus: A Diffusion Tensor Tract-Based Spatial Study

**DOI:** 10.3389/fnins.2021.774638

**Published:** 2021-12-03

**Authors:** Yufeng Tang, Xiaoqin Yuan, Jinfeng Duan, Xianwen Zhang, Jiao Chen, Ying Zhou, Fangzhou Song, Dong Zhou

**Affiliations:** ^1^Department of Neurology, Mianyang Central Hospital, School of Medicine, University of Electronic Science and Technology of China, Mianyang, China; ^2^Department of Radiology, Mianyang Central Hospital, School of Medicine, University of Electronic Science and Technology of China, Mianyang, China; ^3^Basic Medicine College, Chongqing Medical University, Chongqing, China; ^4^Department of Neurology, West China Hospital, Sichuan University, Chengdu, China

**Keywords:** idiopathic normal pressure hydrocephalus (iNPH), tap test, diffusion tensor imaging, tract-based spatial statistics (TBSS), cognitive impairment

## Abstract

The present study was designed to systemically evaluate changes in the diffusion tensor imaging (DTI)-derived parameters of iNPH (idiopathic normal pressure hydrocephalus) patients with different responses to the tap test (TT), and to correlate cognitive impairment with white matter (WM) degeneration. This study included 22 iNPH patients and 14 healthy controls with structural magnetic resonance imaging (MRI) and DTI scanning. DTI was used to explore the differences in fractional anisotropy (FA), mean diffusivity (MD), axial diffusivity (AD), and radial diffusivity (RD) for all participants. DTI parameters were evaluated using an ROI (region of interest)-based and tract-based spatial statistics (TBSS) approach. Neuropsychological assessments and the idiopathic normal pressure hydrocephalus grading scoring scale (iNPHGS) were performed. Compared to the TT non-responders, the TT responders group had significantly lower FA values in the corpus callosum, cingulum cingulate gyrus, superior longitudinal fasciculus, and lower AD values in the right cingulum cingulate gyrus and the left posterior thalamic radiation. Besides, the MD values were significantly increased in the corpus callosum, left anterior corona radiata, and the RD values in the corpus callosum and cingulum cingulate gyrus. In addition, the cognitive improvement was negatively correlated with FA of the corpus callosum, cingulum cingulate gyrus, and MD values of the genu of corpus callosum. While, the cognitive improvement was positively related to the AD of the cingulum cingulate gyrus, superior longitudinal, and RD values of the corpus callosum, cingulum cingulate gyrus and uncinate fasciculus. The ROI specific WM lesions in iNPH patients are the underlying basis for cognitive impairment.

## Introduction

Idiopathic normal pressure hydrocephalus (iNPH) is a complex clinical disease with an undetermined etiology. The clinical characteristics of iNPH include gait disorders, cognitive impairment and urinary incontinence. Ventriculomegaly on neuroimaging and cerebrospinal fluid pressures ranging from 70 to 200 mm H_2_O (1 mm H_2_O = 0.0098 kPa) are primary diagnostic criteria for iNPH (Williams and Malm, 2016). iNPH is one of the few etiologies of reversible dementia. Ventriculo-peritoneal shunting (VPS) is an effective treatment for iNPH (Marmarou et al., 2005) that can significantly improve cognitive function in patients (Klinge et al., 2005; Liu et al., 2016).

The increased aging population across the world has resulted in dementia becoming a major global public health problem. As iNPH is a reversible form of dementia, the disease has become the focus of intense research efforts. The symptoms and neuroimaging findings of iNPH are similar to other neurodegenerative diseases such as Alzheimer’s disease (AD) and Parkinson’s disease (PD) (Kang et al., 2013). All of these clinical entities mainly occur in elderly patients and so iNPH is often found along with other neurodegenerative diseases. According to the uniform diagnostic criteria (Marmarou et al., 2005), the postoperative effects in different iNPH vary significantly. The accurate prediction of the shunt response can distinguish patients with reversible dementia from other forms of the disease.

The tap test (TT) is the most widely used and effective method for the preoperative evaluation of iNPH (Martín-Láez et al., 2016). Patients diagnosed with iNPH show differential responses to the cerebrospinal fluid (Ko et al., 2017). Patients with a positive TT response can obtain obvious improvements in cognitive function after shunt surgery, whilst most TT negative patients usually experience very poor postoperative effects often with no change in cognitive deficits (McKhann and Mayeux, 2010; Wolfsegger and Topakian, 2017). These observations suggest that different mechanisms of cognitive impairment may occur between TT responders and non-responders and could potentially be used to predict cognitive function outcomes after surgery in iNPH patients.

The mechanism of cognitive impairment in iNPH patients remains unclear. The cognitive network is highly complex and its dysfunction in cognitive disorders is an area of intense research interest. Diffusion tensor imaging (DTI) is a magnetic resonance (MR) technique that has recently been used to study white matter (WM) degeneration in patients with iNPH. Amongst the DTI parameters, fractional anisotropy (FA) and mean diffusivity (MD) have been demonstrated as a useful index of WM impairment in iNPH patients (Kanno et al., 2011; Nicot et al., 2014; Radovnický et al., 2016). FA is the most widely used DTI parameter, which reflects the integrity of the axon and is highly sensitive to change in microstructure. MD quantifies cellular and membrane density whereas an increase in MD indicates cellularity, edema, and necrosis of WM (Tae et al., 2018). Previous studies observed lower FA and higher MD within various supratentorial regions including the corticospinal tract (CST), the corpus callosum (CC), and some subcortical WM (Hattori et al., 2011, 2012; Koyama et al., 2013; Daouk et al., 2014). However, few studies have systemically analyzed whole-brain WM microstructures and explored the relationship between the integrity of WM and cognitive decline. The DTI parameters of axial diffusivity (AD) and radial diffusivity (RD) have rarely been reported in previous iNPH studies (Scheel et al., 2012; Jurcoane et al., 2014). RD is a putative myelin marker and increases with demyelination. AD is related to axonal injury and thus decreases in cases of axonal damage (Tae et al., 2018). Furthermore, few studies have compared the differences between TT responders and non-responders in iNPH patients.

This study aimed to systemically evaluate the WM changes in iNPH patients with different responses to the TT, and to correlate cognitive impairment and WM microstructural damage in iNPH patients.

## Materials and Methods

### Participants

A total of 22 patients diagnosed with iNPH in the Neurology Department of Mianyang Central Hospital from May 2016 to December 2019 were included in this study. Before lumbar puncture and at 8, 24, 48, and 72 h after the drainage, gait disturbance, mini-mental state examination (MMSE) score, and the idiopathic normal pressure hydrocephalus grading scoring scale (iNPHGS) were assessed (Tarnaris et al., 2007). Gait improvements at any observation times after drainage, improvements in the MMSE score of ≥3 points, or improvements in the iNPHGS of >1 point were considered a positive criterion for the cerebrospinal fluid discharge test. The twenty-five iNPH patients consisted of 12 patients in the TT responsive group and 13 patients in the TT non-responsive group. A total of 14 control subjects with no cognitive impairments were included in the study across the same period.

### Demographic and Clinical Data Collection

Cognitive function was assessed using the following tests:

1. The MMSE was used to test the subjects’ overall cognitive level including orientation, immediate and short-term memory function, language function, and computational power (Folstein et al., 1975).
2. The digit span test (DST) was used to assess attention and immediate memory in memory function (Richardson, 2007).
3. The verbal fluency test animal (VFT-A) was used to assess working memory and vocabulary storage memory in executive functions, and long-term memory in memory function and semantic smooth function (Carlesimo et al., 1996).
4. The trail-making test A (TMT-A) was used to assess performance functions and attention (O’Leary et al., 1977).
5. The Stroop color-word test-card B (CWT-B) was used to assess attention (Jensen and Rohwer, 1966).
6. The clock drawing test (CDT, Huashan version) was used to assess multiple cognitive functions including the task plan in the executive function, the spatial mechanism function, the semantic and digital memory in the memory function, the abstract thinking ability, and the anti-interference ability (Olazarán et al., 2016).

The iNPHGS is a clinician-rated scale to evaluate the severity of core symptoms of iNPH (cognitive impairment, gait disturbance, and urinary disturbance). The score of each domain ranges from 0 to 4, with higher scores indicating worse symptoms (Tarnaris et al., 2007).

All the subjects were scored at the baseline before the tap test. All of the iNPH patients were scored at 8, 24, 48, and 72 after the tap test.

### Magnetic Resonance Imaging Acquisition and Image Processing

Magnetic resonance imaging was performed on a 3.0T Siemens MAGNETON Skyra using a 12-channel head matrix radio frequency receive coil. The MR imaging protocol included a T1-weighted sequence (TR = 700 ms, TE = 11 ms, 0.9 mm slice separation, giving a voxel size 0.9 mm × 0.9 mm × 0.9 mm), a T2-weighted sequence (TR = 4,910 ms, TE = 99 ms, 5 mm slice separation, giving a voxel size 0.6 mm × 0.6 mm × 5 mm), and a fluid attenuated inversion recovery (FLAIR) sequence (TR = 8000, TE = 99 ms, 5 mm slice separation, giving a voxel size 0.9 mm × 0.9 mm × 5 mm). The DTI data set was acquired by using a spin echo diffusion weighted echo planar imaging sequence with the following parameters: TR = 10,400 ms; TE = 89 ms; FOV = 256 mm × 256 mm; acquisition matrix = 128 × 128; voxel size 2 mm × 2 mm × 2 mm; 75 axial slices; 4 images without (b0) and 60 images with diffusion weighting (b = 1,000 s/mm^–2^) uniformly distributed across 60 gradient directions.

DTI data was processed using several approaches as follows:

a) Tract-based spatial statistics (TBSS): PANDA [Pipeline for Analyzing braiN Diffusion imAges, a MATLAB toolbox which consists of FMRIB Software Library (FSL) and several established packages] was used for the processing of the DTI raw data^1^ (Cui et al., 2013). All of the DTI data of the subjects were automatically processed by TBSS to achieve the DTI scalars FA, MD, AD, and RD used in the analysis.
b) ABA-TBSS: FSL was used to generate a WM map (JHU DTI-based white-matter atlases) by separating all of the whole WM. This approach was used to automatically calculate the average skeleton value of each brain region. The outputs were saved in Excel file format.

Comparison of whole brain WM skeleton (TBSS): quantitative analysis of the whole brain WM skeleton was performed using the built-in TBSS randomize statistical tool in FSL. The statistical results were displayed using the xjview and fslview software packages. The regions of interest (ROIs) were mapped using the JHU DTI-based white-matter atlases: anterior thalamic radiation (ATR), posterior thalamic radiation include optic radiation (PTR), anterior corona radiata (ACR), superior corona radiata (SCR), posterior corona radiata (PCR), tapetum (TAP), cingulum cingulate gyrus (CgC), cingulum hippocampus (CgH), superior fronto-occipital fasciculus (SFOF), inferior frontooccipital fasciculus (IFOF), inferior longitudinal fasciculus (ILF), superior longitudinal fasciculus (SLF), superior longitudinal fasciculus temporal part (SLFT), sagittal stratum (include ILF and IFOF) (SS), and uncinate fasciculus (UF) within each hemisphere, and forceps major (F-major), forceps minor (F-minor), genu of corpus callosum (GCC), body of corpus callosum (BCC), splenium of corpus callosum (SCC), and fornix (column and body of fornix) (FN) across hemispheres (shown in Supplementary Figure 1) (Alexander et al., 2007).

### Statistical Analysis

Statistical analysis was performed using SPSS 20.0 software. A *p*-value threshold of <0.05 was used to determine the level of statistical significance. The demographic data, neuropsychological scores, and the iNPHGS scores were presented as the mean ± standard deviation. One-way analysis of variance (ANOVA) was used to compare demographic data and baseline cognitive scores among the control group and the iNPH patients (TT responsive and TT non-responsive groups). A Mann–Whitney *U* test was used to compare the maximum improvement scores (time duration) of the neuropsychological performance in the two iNPH groups after the TT. Comparison of the average skeletal values (FA, MD, AD, and RD) in the ROIs was performed using ANOVA among different groups. Bonferroni correction was used to control for multiple comparisons, while uncorrected results are also presented because Bonferroni’s correction is quite conservative (Narum, 2006). Correlation analysis was performed between the DTI parameters (FA, MD, AD, and RD) and the MMSE scores, the total cognitive scores, and the improvement of cognitive scores by Pearson correlation analysis. The Pearson coefficient (*r*-value) > 0.4 and *P* < 0.05 were set to define moderate correlation.

## Results

### Demographic and Clinical Profiles

The detailed demographic and clinical information from the patients is presented in Table 1. This study involved 22 patients with iNPH who met the inclusion criteria and 14 healthy controls (HCs). In the iNPH patient group, 20 patients were male and 2 patients were female. The average age of the patients in this group was 75.40 ± 5.83 years and the average education period was 6.92 ± 5.72 years. The control group consisted of 11 males and 3 females with an average age of 75.36 ± 5.76 years and an average education period of 6.43. ± 4.69 years. There were no significant differences in gender, age, and years of schooling between the iNPH patients and the HCs. The iNPH patients had significantly poorer performance in the MMSE, DST, VFT-A, CWT-B, TMT-A, and CDT compared to the HCs (*P* < 0.05). According to the improvements after the TT, 12 patients were classified in the TT responsive group (TT-R) and 13 patients were classified in the TT non-responsive group (TT-nR). No significant differences were found between the TT-R and TT-nR groups in age, sex, years of schooling, and baseline cognitive levels (MMSE, DST, VFT-A, CWT-B, TMT-A, and CDT scores). The iNPHGS score of the TT-R group was higher than the TT-nR group suggesting that the clinical symptoms were more severe in the TT-R group.

**TABLE 1:** Demographic and characteristics of all subjects.

Parameter	TT-R	TT-nR	Controls	*P*-value^1^	*P*-value^2^
Number, *n*	10	12	14	–	–
Age, y	76.10 ± 4.15	74.41 ± 7.53	75.18 ± 5.76	0.93	0.52
Gender, M/F	10/0	11/1	11/3	0.36	1
Education, y	6.50 ± 6.10	7.50 ± 5.93	7.05 ± 4.69	0.74	0.70
iNPHGS	7.90 ± 2.08	5.50 ± 1.31		–	0.00
MMSE	16.30 ± 7.45	19.42 ± 5.50	24.57 ± 2.59	0.00	0.27
DST	5.90 ± 1.66	6.17 ± 1.75	9.79 ± 1.48	0.00	0.72
VFT-A	6.90 ± 2.08	7.08 ± 2.35	13.36 ± 2.56	0.00	0.85
CWT-B	38.40 ± 8.49	42.67 ± 6.96	47.14 ± 2.60	0.00	0.21
TMT-A	11.58 ± 8.84	8.23 ± 8.37	0.93 ± 1.27	0.00	0.46
CDT	8.95 ± 8.76	11.15 ± 9.87	22.36 ± 7.82	0.00	0.55

*^1^Comparison between all iNPH patients (TT-R and TT-nR) and control subjects.*

*^2^Comparison between TT-R and TT-nR patients. TT, tap test; TT-R, TT responsive group; TT-nR, TT non-responsive group; MMSE, Mini-Mental State Examination; DST, Digit Span forward; VFT-A, Verbal Fluency Test –ANIMAL; TMT-A, Trail Making Test A; CDT, Clock Drawing Test; CWT-B, Stroop Color Word Test- card B.*

### Tract-Based Spatial Statistics Whole Brain White Matter Skeleton Comparison

**FIGURE 1:**
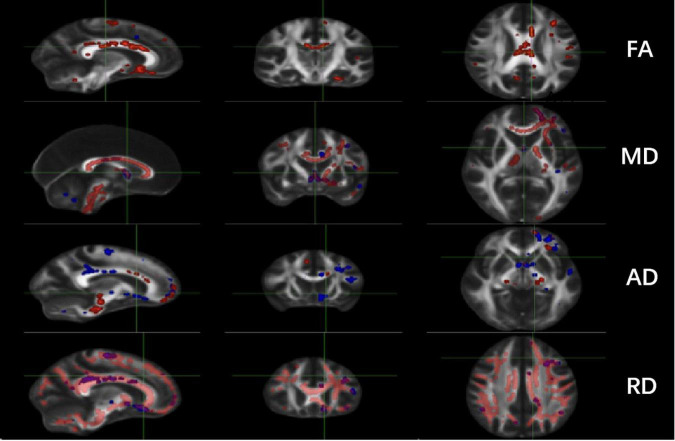
Comparisons of DTI imaging in group analyses of TBSS. Results of TBSS between TT responsive group and TT non-responsive group. Significant region (*P* < 0.05) illustrated in warm colors for decreased values and in cool colors for increased values on mean WM skeleton. DTI, diffusion tensor imaging; TBSS, tract-based spatial statistics; FA, fractional anisotropy; MD, mean diffusivity; AD, axial diffusivity; RD, radial diffusivity.

Significant changes were observed in the TT-R group compared to the TT-nR group. These included decreases in the FA skeleton values in specific areas (GCC, BCC, SCC, F-major, FN, CgC, L-CgH, L-ATR, L-IFOF, SLF, L-UF) (*P* < 0.05), and decreased in the MD skeleton values in areas of the CC, FN, B-CgC, B-PTR, L-CgH, L-IFOF, SLF, L-UF (*P* < 0.05), AD values in areas of the GCC, BCC, SCC, F-major, CgC, PTR, IFOF (*P* < 0.05) and RD values in areas of the CC, CgC, CgH, PTR, IFOF, ILF, ACR, UF (*P* < 0.05) (shown in Figure 1).

### The Region of Interest Average Skeleton Values Based on ABA-Tract-Based Spatial Statistics Comparison

Comparison of the TT-R and TT nR groups showed that the average FA skeleton values in the areas of the GCC, BCC, SCC, F-major, CgC, SLF, L-SLFT were significantly lower in the TT-R group (*P* < 0.05). The average MD skeleton values in the areas of the GCC, BCC, L-ACR, and the average RD skeleton values in the areas of GCC, BCC, SCC, CgC, L-SLFT, L-ACR were significantly increased in the TT-R group (*P* < 0.05). Also, the average AD skeleton values in the areas of the R-CgC, L-PTR, L-SLFT were significantly reduced in the TT-R group (*P* < 0.05) (Shown in Table 2 and Supplementary Table 1).

**TABLE 2:** ABA-TBSS analysis results.

	TT-R vs. HC				TT-nR vs. HC					TT-R vs. TT-nR			
ROI	FA	MD	AD	RD	FA	MD	AD	RD		FA	MD	AD	RD
ATR-L													
ATR-R										↓			
CgC-L	↓			↑						↓			↑
CgC-R	↓		↓	↑	↓		↓	↑		↓		↓	↑
CgH-L				↑									
CgH-R													
F-major	↓			↑	↓			↑					
GCC	↓	↑	↑	↑	↓	↑	↑	↑		↓	↑		↑
BCC	↓	↑		↑						↓	↑		↑
SCC	↓	↑		↑	↓	↑		↑		↓			↑
FN	↓												
ACR-R	↓	↑	↑	↑		↑	↑	↑			↑		
ACR-L	↓	↑	↑	↑		↑	↑				↑	↑	↑
F-minor	↓	↑		↑	↓	↑	↑	↑		↓			
IFOF-L	↓	↑		↑	↓	↑		↑					
IFOF-R	↓			↑	↓	↑		↑					
ILF-L	↓				↓								
ILF-R	↓			↑	↓								
SLF-L	↓			↑	↓	↑			↑			↓	
SLF-R	↓			↑	↓				↑	↓			
UF-L	↓	↑		↑									
UF-R													
SLFT-L	↓			↑						↓			
SLFT-R												↓	
SCR-L		↑	↑	↑			↑						↑
SCR-R		↑	↑				↑						
PCR-R						↑	↑						
PCR-L PTR-R													
PTR-L	↓		↓		↓				↑			↓	
SS-R	↓			↑		↑							
SS-L	↓			↑	↓	↑			↑				
SFOF-R	↓		↑				↑						
SFOF-L			↑		↑		↑						
TAP-R													
TAP-L													

*Up arrows (↑) indicate higher values, and down arrows (↓) indicate lower values in the former group compared with the later group.*

*TT, tap test; TT-R, TT responsive group; TT-nR, TT non-responsive group; HC, healthy controls; TBSS, tract-based spatial statistics; FA, fractional anisotropy; MD, mean diffusivity; AD, axial diffusivity; RD, radial diffusivity; L-ATR, anterior thalamic radiation L; R-ATR, anterior thalamic radiation R; L-PTR, posterior thalamic radiation include optic radiation L; R-PTR, posterior thalamic radiation include optic radiation R; L-ACR, anterior corona radiata R; R-ACR, anterior corona radiata L; L-SCR, superior corona radiata R; R-SCR, superior corona radiata L; L-PCR, posterior corona radiata R; R-PCR, posterior corona radiata L; F-major, forceps major; F-minor, forceps minor; GCC, genu of corpus callosum; BCC, body of corpus callosum; SCC, splenium of corpus callosum; L-TAP, tapetum L; R-TAP, tapetum R; FN, fornix (column and body of fornix); L-CgC, cingulum cingulate gyrus L; R-CgC, cingulum cingulate gyrus R; L-CgH, cingulum hippocampus L; R-CgH, cingulum hippocampus R; L-SFOF, superior fronto-occipital fasciculus L; R-SFOF, superior fronto-occipital fasciculus R; L-IFOF, inferior frontooccipital fasciculus L; R-IFOF, inferior frontooccipital fasciculus R; L-ILF, inferior longitudinal fasciculus L; R-ILF, inferior longitudinal fasciculus R; L-SLF, superior longitudinal fasciculus L; R-SLF, superior longitudinal fasciculus R; L-SLFT, superior longitudinal fasciculus temporal part L; R-SLFT, superior longitudinal fasciculus temporal part R; L-SS, sagittal stratum (include ILF and IFOF) L; R-SS, sagittal stratum (include ILF and IFOF) R; L-UF, uncinate fasciculus L; R-UF, uncinate fasciculus R.*

### Correlation Analysis Between the Region of Interest Average Skeleton Values and Cognitive Performance in Idiopathic Normal Pressure Hydrocephalus Patients

#### Correlation Analysis Between Region of Interest Average Skeleton Values (Fractional Anisotropy, Mean Diffusivity, Axial Diffusivity, and Radial Diffusivity) and Baseline Total Cognitive Scores in Idiopathic Normal Pressure Hydrocephalus Patients

The total cognitive scores of the iNPH patients were positively correlated with the average FA values of the GCC, BCC, SCC, F-major, F-minor, CgC, and the ILF (*r* > 0.4, *P* < 0.05). The total cognitive scores were negatively correlated with the average MD values of the GCC, BCC, SCC, F-major, F-minor, ACR, and SCR (*r* > 0.4, *P* < 0.05), the total cognitive scores were negatively correlated with the average AD values of the GCC and SCR (*r* > 0.4, *P* < 0.05) and positively correlated with the average AD values of the CgC (*r* > 0.4, *P* < 0.05). The total cognitive scores were negatively correlated with the average RD values of the GCC, BCC, SCC, F-major, F-minor, CgC, SLF, and ACR (*r* > 0.4, *P* < 0.05) (shown in Table 3 and Supplementary Table 2).

**TABLE 3:** Correlation analysis between the ROI average skeleton values and cognitive performance in iNPH patients.

	TS				MMSE				CI			
ROI	FA	MD	AD	RD	FA	MD	AD	RD	FA	MD	AD	RD
ATR-L												
ATR-R												
CgC-L	0.353			−0.380					−0.680			0.547
CgC-R	0.533		0.427	−0.479	0.441			−0.412	−0.591		−0.472	
CgH-L		−0.368		−0.395								
CgH-R												
F-major	0.546	−0.451		−0.522	0.436	−0.360		−0.418				
F-minor	0.412	−0.456	−0.351	−0.458		−0.373		−0.377	−0.531			
IFOF-L												
IFOF-R												
LIF-L												
ILF-R	0.411											
SLF-L	0.389	−0.380		−0.420					−0.473		−0.519	
SLF-R				−0.353								
UF-L												
UF-R												
SLFT-L									−0.527			
SLFT-R											−0.557	
GCC	0.446	−0.576	−0.493	−0.540	0.389	−0.524	−0.439	−0.495	−0.608			0.531
BCC	0.430	−0.508	−0.362	−0.509	0.433	−0.507	−0.346	−0.514	−0.606	0.475		0.574
SCC	0.512	−0.537		−0.541	0.394	−0.398		−0.413	−0.496			
FN												
ACR-R		−0.400	−0.380	−0.400								
ACR-L		−0.354	−0.386								0.463	
SCR-R												
SCR-L		−0.446	−0.525	−0.368		−0.401	−0.447	−0.345				
PCR-R		−0.343	−0.360									
PCR-L												
PTR-R												
PTR-L	0.348										−0.503	
SS-R			−0.149	−0.336							−0.497	
SS-L												
SFOF-R												
SFOF-L			−0.396									
TAP-R												
TAP-L												

*Only significant correlations were displayed, and more detailed information were shown in the Supplementary Materials.*

*TS, Total scores; CI, cognitive improvement; MMSE, Mini-Mental State Examination; FA, fractional anisotropy; MD, mean diffusivity; AD, axial diffusivity; RD, radial diffusivity; L-ATR, anterior thalamic radiation L; R-ATR, anterior thalamic radiation R; L-PTR, posterior thalamic radiation include optic radiation L; R-PTR, posterior thalamic radiation include optic radiation R; L-ACR, anterior corona radiata R; R-ACR, anterior corona radiata L; L-SCR, superior corona radiata R; R-SCR, superior corona radiata L; L-PCR, posterior corona radiata R; R-PCR, posterior corona radiata L; F-major, forceps major; F-minor, forceps minor; GCC, genu of corpus callosum; BCC, body of corpus callosum; SCC, splenium of corpus callosum; L-TAP, tapetum L; R-TAP, tapetum R; FN, fornix (column and body of fornix); L-CgC, cingulum cingulate gyrus L; R-CgC, cingulum cingulate gyrus R; L-CgH, cingulum hippocampus L; R-CgH, cingulum hippocampus R; L-SFOF, superior fronto-occipital fasciculus L; R-SFOF, superior fronto-occipital fasciculus R; L-IFOF, inferior frontooccipital fasciculus L; R-IFOF, inferior frontooccipital fasciculus R; L-ILF, inferior longitudinal fasciculus L; R-ILF, inferior longitudinal fasciculus R; L-SLF, superior longitudinal fasciculus L; R-SLF, superior longitudinal fasciculus R; L-SLFT, superior longitudinal fasciculus temporal part L; R-SLFT, superior longitudinal fasciculus temporal part R; L-SS, sagittal stratum (include ILF and IFOF) L; R-SS, sagittal stratum (include ILF and IFOF) R; L-UF, uncinate fasciculus L; R-UF, uncinate fasciculus R.*

#### Correlation Between the Region of Interest Average Skeleton Values (Fractional Anisotropy, Mean Diffusivity, Axial Diffusivity, and Radial Diffusivity) and Cognitive Improvements After the Tap Test in Idiopathic Normal Pressure Hydrocephalus Patients

A moderate negative correlation was observed between the cognitive improvement and the mean FA of GCC, BCC, SCC, F-minor, CgC, SLF, and SLFT in iNPH patients after lumbar puncture (*r* > 0.4, *P* < 0.05), and also with the mean MD of GCC (*r* > 0.4, *P* < 0.05). The degree of cognitive improvement in iNPH patients was positively related to the mean AD of the CgC, SLF, SLFT, and SS (*r* > 0.4, *P* < 0.05), and also with the average RD values of the GCC, BCC, SCC, CgC, ATR, and UF (*r* > 0.4, *P* < 0.05) (shown in Table 3 and Supplementary Material 2).

## Discussion

The underlying mechanisms of cognitive impairment have been the major research efforts in the field of iNPH research. The TT is the most widely used and effective method for preoperative evaluation of iNPH and the test is used to clinically classify patients into two groups. Patients who respond to the TT can achieve improvements in cognitive performance after shunt surgery, whilst patients who are not responsive to the TT usually experience very poor postoperative effects. These observations suggest different mechanisms of cognitive impairment between TT-R and TT-nR patients.

The present study used the TBSS method and a quantitative ROI analysis of skeletonized brain maps to compare differences in the cognitive-related WM microstructure of iNPH patients with different TT responses. Our data showed that the microstructural WM damage in TT-R patients was significantly more severe than in TT-nR patients. Furthermore, we assessed the associations between FA, MD, AD, and RD values and the cognitive performance of iNPH patients.

The mean FA in the areas of the GCC, BCC, SCC, F-major, FN, B-CgC, L-CgH, L-ATR, L-IFOF, SLF, and L-UF were significantly lower in the TT responsive compared to the TT non-responsive group (*P* < 0.05). Also, the MD and RD in the area of the GCC, BCC, SCC, B-CgC, and ATR were significantly increased (*P* < 0.05). These results indicated that WM edema or the destruction of myelin sheath were more severe in the TT-R group than in the TT-nR group.

In comparison to the subjects in the HC group, patients in the TT-R and TT-nR groups had more extensive microstructural damage presenting with lower FA, higher MD, and RD in CC, CgC, ATR, ACR, SLF, and UF. However, no significant difference was found in preoperative cognitive performance between the TT-R and TT-nR groups. These data may indicate that the cognitive dysfunction in the TT-R group is mostly caused by WM injury, whilst cortical volume atrophy plays a more contributing role in the cognitive decline of patients in the TT-nR group. This hypothesis is supported by previous studies. Kang et al. (2013) found that CSFTT non-responders had statistically significant cortical thinning in the left superior frontal gyrus compared to responders suggesting that comorbid AD pathology might be related to the cortical thinning patterns found in CSFTT non-responders. Also, biopsy studies found that iNPH patients with pathological evidence of AD exhibited more severe initial symptoms and had lower shunt responsiveness compared to patients without AD (Golomb et al., 2000; Savolainen et al., 2002; Picascia et al., 2016).

Correlation analysis showed significant associations between lower FA, higher MD, higher AD, higher RD and poor executive performance in the GCC, BCC, SCC, F-major, and F-minor. The data indicated that the corpus callosum and cingulate gyrus are involved in both memory and executive function, furthermore, different parts of the corpus callosum may participate in different cognitive functions.

The data presented in this study are compatible with other previous studies (Bettcher et al., 2016). Our data showed that WM damage is dominated by the anterior and superior part of the lateral ventricle in iNPH patients, particularly in the frontal lobe, which may account for the prominent executive impairment of iNPH patients. Also, our results showed that more severe damage in the anterior, outer, and upper regions of the periventricular WM obtains more obvious cognitive improvement after cerebrospinal fluid drainage in patients with iNPH. Particularly, the RD values in the corpus callosum and cingulate gyrus were significantly associated with cognitive improvement suggesting that the edema and WM degeneration in the area of anterior and superior lateral ventricles were reversible. WM demyelination, wallerian degeneration, and late axonal degeneration are short-term irreversible processes. In contrast, edema and early axonal degeneration of WM are reversible pathologies. We speculate that the TT rapidly reduces the extravasation pressure of the cerebrospinal fluid by releasing cerebrospinal fluid, and subsequently reduces edema in the periventricular WM. The changes significantly improve cognitive function in iNPH patients. However, the extent of WM fiber stretching in this area is not related to the degree of cognitive improvement after drainage. Our findings may suggest that edema in the area of anterior and superior lateral ventricles contributed mostly to the reversible cognitive impairment.

Overall, these observations confirm the role of the hydrocephalus effect in the occurrence of reversible cognitive impairment in iNPH patients.

This study had several limitations as the analysis was performed on a relatively small sample size and further validation is required in larger patient cohorts. Also, due to the low acceptance rate of shunt surgery for iNPH patients in the Chinese population, our study lacked postoperative follow-up data to further validate the longer-term responses of patients. Prospective cohort studies need to be designed to confirm the values of DTI parameters as a non-invasive imaging biomarker to predict post-operative cognitive improvement in iNPH patients. At last, combining more scales of information, such as radiomics features, might lead to more fine-grained findings in the future.

## Conclusion

1. The extensive microstructural damage of cognitive WM in iNPH patients is the material basis for the development of cognitive impairment.
2. The microstructural damage of the anterior superior ventricle and the WM in the frontal ventricle in TT positive iNPH patients was greater than TT negative patients.
3. The microstructural changes of the CC, the cingulate ligament and the adjacent radiant fibers can affect the memory and executive functions in the cognitive field of iNPH patients, whilst microstructural changes of the anterior subcortical WM in the frontal lobe mainly affect the executive features.
4. The more severe the edema degeneration of WM in the anterior superior region of the lateral ventricle, the more obvious the cognitive improvement in the iNPH patients after the TT. The decrease in the WM FA value and increase of RD value in this region has diagnostic and prognostic value in iNPH patients.

## Data Availability Statement

The raw data supporting the conclusions of this article will be made available by the authors, without undue reservation, to any qualified researcher.

## Ethics Statement

The studies involving human participants were reviewed and approved by The Ethics Committee of Mianyang Central Hospital. The patients/participants provided their written informed consent to participate in this study.

## Author Contributions

YT and DZ: conceptualization. JC, YZ, and YT: methodology. XZ and JD: project administration. FS and DZ: supervision. XY: writing – original draft. YT: writing – review and editing. All authors contributed to the article and approved the submitted version.

## Conflict of Interest

The authors declare that the research was conducted in the absence of any commercial or financial relationships that could be construed as a potential conflict of interest.

## Publisher’s Note

All claims expressed in this article are solely those of the authors and do not necessarily represent those of their affiliated organizations, or those of the publisher, the editors and the reviewers. Any product that may be evaluated in this article, or claim that may be made by its manufacturer, is not guaranteed or endorsed by the publisher.

## Footnotes

1
http://www.nitrc.org/projects/panda/

## Abbreviations

DTI: diffusion tensor imaging
TBSS: tract-based spatial statistics
WM: white matter
FA: fractional anisotropy
MD: mean diffusivity
AD: axial diffusivity
RD: radial diffusivity
TT: tap test
TT-R: TT responsive group
TT-nR: TT non-responsive group
MMSE: Mini-Mental State Examination
DST: digit span forward
VFT-A: Verbal Fluency Test –ANIMAL
TMT-A: Trail Making Test A
CDT: Clock Drawing Test
CWT-B: Stroop Color Word Test- card B
L-ATR: anterior thalamic radiation L
R-ATR: anterior thalamic radiation R
L-PTR: posterior thalamic radiation include optic radiation L
R-PTR: posterior thalamic radiation include optic radiation R
L-ACR: anterior corona radiata R
R-ACR: anterior corona radiata L
L-SCR: superior corona radiata R
R-SCR: superior corona radiata L
L-PCR: posterior corona radiata R
R-PCR: posterior corona radiata L
F-major: forceps major
F-minor: forceps minor
GCC: genu of corpus callosum
BCC: body of corpus callosum
SCC: splenium of corpus callosum
L-TAP: tapetum L
R-TAP: tapetum R
FN: fornix (column and body of fornix)
L-CgC: cingulum cingulate gyrus L
R-CgC: cingulum cingulate gyrus R
L-CgH: cingulum hippocampus L
R-CgH: cingulum hippocampus R
L-SFOF: superior fronto-occipital fasciculus L
R-SFOF: superior fronto-occipital fasciculus R
L-IFOF: inferior frontooccipital fasciculus L
R-IFOF: inferior frontooccipital fasciculus R
L-ILF: inferior longitudinal fasciculus L
R-ILF: inferior longitudinal fasciculus R
L-SLF: superior longitudinal fasciculus L
R-SLF: superior longitudinal fasciculus R
L-SLFT: superior longitudinal fasciculus temporal part L
R-SLFT: superior longitudinal fasciculus temporal part R
L-SS: sagittal stratum (include ILF and IFOF) L
R-SS: sagittal stratum (include ILF and IFOF) R
L-UF: uncinate fasciculus L
R-UF: uncinate fasciculus R.
