# Supplementary material for: White Matter Characteristics of Cognitive Impairment in Tap-Test Positive Idiopathic Normal Pressure Hydrocephalus: A Diffusion Tensor Tract-Based Spatial Study
Source: Front Neurosci. 2021 Dec 3;15:774638. doi: 10.3389/fnins.2021.774638 (PMC8678068; doi:10.3389/fnins.2021.774638)
Supplement: Supplementary file 1 [file Data_Sheet_1.pdf]

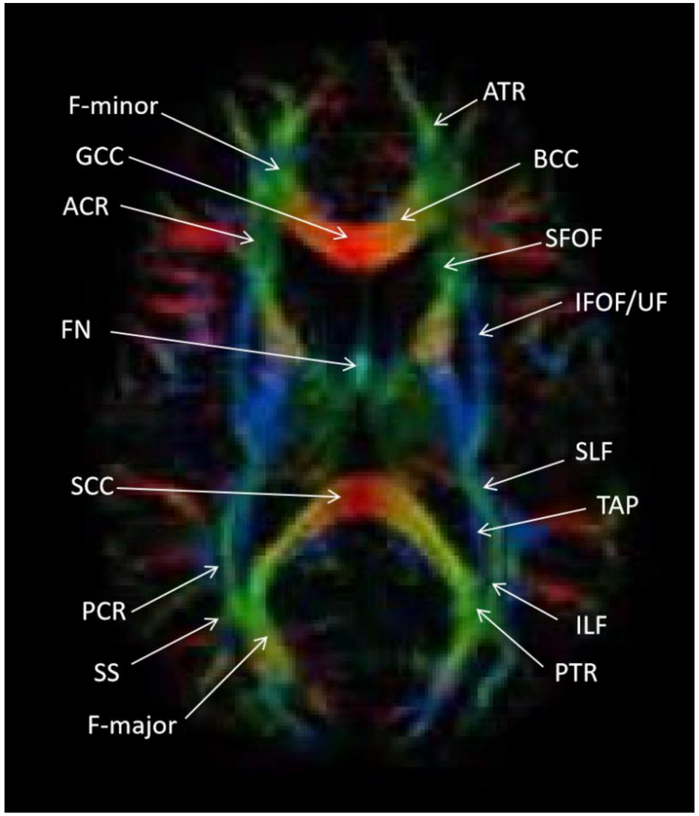

Figure S1. Illustration of ROIs on the DTI images. ATR: anterior thalamic radiation, PTR: posterior thalamic radiation include optic radiation, ACR: anterior corona radiata, SCR: superior corona radiata, PCR: posterior corona radiata, TAP: tapetum, SFOF: superior fronto-occipital fasciculus, IFOF: inferior frontooccipital fasciculus, ILF: inferior longitudinal fasciculus, SLF: superior longitudinal fasciculus, SS: sagittal stratum（include ILF and IFOF）, UF uncinate fasciculus, F-major: forceps major, F-minor: forceps minor, GCC: genu of corpus callosum, BCC: body of corpus callosum, SCC: splenium of corpus callosum, FN: fornix（column and body of fornix)

Table S1: The detailed data for ABA-TBSS analysis results.

	TT-R VS TT-nR				TT-R VS HC				TT-nR VS HC			
ROI	**FA**	**MD**	**AD**	**RD**	**FA**	**MD**	**AD**	**RD**	**FA**	**MD**	**AD**	**RD**
L-ATR	0.219	0.454	0.697	0.357	0.451	0.249	0.145	0.329	0.567	0.674	0.245	0.976
BCC	0.003	0.011	0.369	0.003	0.000	0.000	0.171	0.000	0.068	0.136	0.621	0.07
F-major	0.206	0.346	0.929	0.259	0.000	0.012	0.065	0.001	0.001	0.079	0.057	0.008
F-minor	0.018	0.519	0.371	0.176	0.000	0.002	0.249	0.000	0.014	0.007	0.028	0.006
FN	0.09	0.512	0.876	0.415	0.017	0.369	0.834	0.208	0.435	0.808	0.687	0.642
GCC	0.002	0.04	0.847	0.006	0.000	0.000	0.048	0.000	0.043	0.008	0.02	0.014
L-ACR	0.114	0.013	0.011	0.018	0.007	0.000	0.000	0.000	0.208	0.049	0.025	0.075
L-CgC	0.000	0.118	0.217	0.002	0.000	0.009	0.191	0.000	0.052	0.228	0.968	0.074
L-CgH	0.13	0.27	0.758	0.06	0.097	0.059	0.526	0.017	0.91	0.389	0.304	0.588
L-IFOF	0.554	0.975	0.252	0.693	0.002	0.022	0.651	0.005	0.007	0.012	0.081	0.009
L-ILF	0.839	0.631	0.302	0.874	0.035	0.326	0.856	0.122	0.038	0.113	0.336	0.066
L-PCR	0.733	0.52	0.231	0.763	0.099	0.321	0.69	0.191	0.154	0.078	0.08	0.082

L-PTR	0.954	0.219	0.021	0.554	0.009	0.985	0.01	0.204	0.004	0.176	0.818	0.046
L-SCR	0.547	0.324	0.394	0.319	0.799	0.01	0.001	0.042	0.689	0.072	0.006	0.251
L-SFOF	0.069	0.442	0.9	0.235	0.656	0.138	0.016	0.418	0.014	0.44	0.006	0.644
L-SLF	0.016	0.601	0.028	0.606	0.000	0.123	0.262	0.005	0.006	0.027	0.19	0.012
L-SLFT	0.013	0.151	0.817	0.034	0.000	0.156	0.341	0.013	0.186	0.945	0.437	0.717
L-SS	0.936	0.422	0.154	0.675	0.03	0.17	0.875	0.049	0.015	0.021	0.153	0.011
L-TAP	0.874	0.795	0.63	0.916	0.336	0.326	0.462	0.274	0.384	0.178	0.185	0.193
L-UF	0.615	0.779	0.949	0.716	0.044	0.041	0.126	0.04	0.098	0.055	0.112	0.064
R-ACR	0.167	0.536	0.92	0.394	0.009	0.006	0.017	0.005	0.165	0.019	0.014	0.027
R-ATR	0.047	0.466	0.916	0.268	0.403	0.147	0.119	0.18	0.174	0.439	0.072	0.823
R-CgC	0.000	0.43	0.012	0.021	0.000	0.07	0.000	0.000	0.000	0.264	0.014	0.008
R-CgH	0.951	0.351	0.089	0.694	0.7	0.744	0.649	0.452	0.626	0.166	0.151	0.212
R-IFOF	0.469	0.809	0.086	0.786	0.011	0.091	0.452	0.021	0.042	0.038	0.256	0.026
R-ILF	0.442	0.919	0.27	0.685	0.008	0.136	0.94	0.039	0.033	0.085	0.19	0.069
R-PCR	0.888	0.473	0.277	0.621	0.107	0.251	0.451	0.182	0.059	0.045	0.047	0.05
R-PTR	1	0.615	0.341	0.807	0.048	0.717	0.1	0.209	0.032	0.341	0.455	0.105

R-SCR	0.641	0.301	0.292	0.341	0.878	0.021	0.005	0.059	0.722	0.153	0.043	0.306
R-SFOF	0.205	0.561	0.807	0.475	0.373	0.028	0.005	0.071	0.642	0.076	0.006	0.231
R-SLF	0.023	0.936	0.166	0.334	0.000	0.062	0.438	0.003	0.017	0.053	0.468	0.02
R-SLFT	0.56	0.231	0.026	0.665	0.351	0.777	0.175	0.79	0.715	0.299	0.273	0.44
R-SS	0.372	0.815	0.061	0.627	0.018	0.1	0.777	0.027	0.098	0.044	0.071	0.057
R-TAP	0.386	0.405	0.389	0.434	0.475	0.97	0.626	0.741	0.086	0.331	0.659	0.22
R-UF	0.229	0.627	0.813	0.451	0.584	0.511	0.208	0.741	0.445	0.213	0.268	0.233
SCC	0.001	0.135	0.325	0.018	0.000	0.001	0.645	0.000	0.002	0.023	0.543	0.007

Table S2: The detailed data for correlation analysis between the ROI average skeleton values and cognitive performance in iNPH patients.

	**Total scores**								**MMSE**								**Cognitive improvement**							
ROI	**FA**		**MD**		**AD**		**RD**		**FA**		**MD**		**AD**		**RD**		**FA**		**MD**		**AD**		**RD**	
	r	p	r	p	r	p	r	p	r	p	r	p	r	p	r	p	r	p	r	p	r	p	r	p
L-ATR	-0.131	0.454	-0.063	0.72	-0.169	0.33	-0.007	0.97	-0.153	0.381	-0.02	0.911	-0.119	0.498	0.032	0.856	-0.171	0.458	0.157	0.497	0.068	0.769	0.191	0.407
R-ATR	0.019	0.915	-0.16	0.358	-0.187	0.281	-0.139	0.425	0.058	0.739	-0.154	0.377	-0.179	0.304	-0.134	0.442	-0.305	0.178	0.121	0.603	-0.025	0.913	0.185	0.421

L-CgC	0.353*	0.037	-0.317	0.064	-0.013	0.94	-0.380*	0.024	0.24	0.165	-0.281	0.102	-0.066	0.705	-0.306	0.074	-0.680**	0.001	0.245	0.284	-0.314	0.165	0.547*	0.01
R-CgC	0.533**	0.001	-0.253	0.142	0.427*	0.01	-0.479**	0.004	0.441**	0.008	-0.247	0.153	0.311	0.069	-0.412*	0.014	-0.591**	0.005	0.127	0.582	-0.472*	0.031	0.41	0.065
L-CgH	0.224	0.197	-.368*	0.03	-0.211	0.224	-0.395*	0.019	0.19	0.275	-0.306	0.074	-0.191	0.271	-0.317	0.064	-0.269	0.239	0.217	0.344	-0.023	0.923	0.331	0.143
R-CgH	0.096	0.583	-0.02	0.911	0.128	0.463	-0.107	0.54	0.191	0.273	-0.062	0.723	0.102	0.56	-0.156	0.371	0.055	0.814	-0.223	0.331	-0.363	0.106	-0.122	0.598
F-major	0.546**	0.001	-0.451**	0.007	0.152	0.384	-0.522**	0.001	0.436**	0.009	-0.360*	0.033	0.123	0.48	-0.418*	0.012	-0.185	0.423	0.105	0.652	-0.076	0.744	0.149	0.52
F-minor	0.412*	0.014	-0.456**	0.006	-0.351*	0.038	-0.458**	0.006	0.333	0.051	-0.373*	0.027	-0.283	0.1	-0.377*	0.026	-0.531*	0.013	0.113	0.625	-0.214	0.351	0.278	0.222
L-IFOF	0.251	0.146	-0.282	0.101	-0.228	0.187	-0.281	0.102	0.13	0.458	-0.155	0.375	-0.127	0.467	-0.154	0.377	-0.003	0.989	-0.11	0.637	-0.286	0.208	-0.032	0.892
R-IFOF	0.259	0.133	-0.235	0.175	-0.023	0.895	-0.277	0.107	0.159	0.362	-0.125	0.473	0.045	0.796	-0.165	0.344	-0.057	0.806	-0.139	0.549	-0.389	0.081	-0.041	0.861
L-ILF	0.291	0.09	-0.234	0.177	-0.129	0.459	-0.273	0.113	0.172	0.322	-0.111	0.524	-0.048	0.786	-0.137	0.432	0.035	0.879	-0.245	0.285	-0.373	0.096	-0.163	0.481
R-ILF	0.411*	0.014	-0.248	0.151	-0.038	0.826	-0.328	0.054	0.327	0.055	-0.136	0.435	0.059	0.737	-0.217	0.21	-0.015	0.949	-0.199	0.386	-0.364	0.105	-0.103	0.656
L-SLF	0.389*	0.021	-0.380*	0.024	-0.198	0.254	-0.420*	0.012	0.274	0.111	-0.255	0.139	-0.124	0.478	-0.287	0.095	-0.473*	0.03	-0.192	0.406	-0.519*	0.016	0.047	0.838
R-SLF	0.316	0.064	-0.321	0.06	-0.138	0.428	-0.353*	0.038	0.231	0.182	-0.234	0.176	-0.102	0.561	-0.257	0.137	-0.389	0.081	-0.097	0.677	-0.369	0.1	0.087	0.709
L-UF	0.097	0.581	-0.204	0.239	-0.265	0.124	-0.15	0.391	-0.004	0.984	-0.075	0.667	-0.154	0.378	-0.028	0.874	-0.005	0.982	0.039	0.868	0.08	0.73	0.009	0.969
R-UF	-0.158	0.364	0.001	0.994	-0.098	0.577	0.043	0.806	-0.128	0.465	0.048	0.785	-0.001	0.995	0.064	0.715	0.263	0.249	-0.09	0.697	0.075	0.747	-0.149	0.518
L-SLFT	0.183	0.292	0.066	0.705	0.237	0.171	-0.04	0.821	0.069	0.692	0.152	0.384	0.266	0.122	0.059	0.738	-0.527*	0.014	0.21	0.36	-0.165	0.474	0.386	0.084
R-SLFT	-0.053	0.764	-0.049	0.779	-0.146	0.404	0.009	0.96	-0.043	0.805	0.003	0.988	-0.062	0.724	0.032	0.853	-0.08	0.731	-0.338	0.134	-0.557**	0.009	-0.153	0.507

GCC	0.446**	0.007	-0.576**	0.001	-0.493**	0.003	-0.540**	0.001	0.389*	0.021	-0.524**	0.001	-0.439**	0.008	-0.495**	0.002	-0.608**	0.003	0.396	0.075	-0.07	0.763	0.531*	0.013
BCC	0.430**	0.01	-0.508**	0.002	-0.362*	0.033	-0.509**	0.002	0.433**	0.009	-0.507**	0.002	-0.346*	0.042	-0.514**	0.002	-0.606**	0.004	0.475*	0.03	0.154	0.506	0.574**	0.007
SCC	0.512**	0.002	-0.537**	0.001	-0.262	0.128	-0.541**	0.001	0.394*	0.019	-0.398*	0.018	-0.157	0.369	-0.413*	0.014	-0.496*	0.022	0.201	0.382	-0.27	0.236	0.356	0.113
FN	0.222	0.201	-0.119	0.497	-0.018	0.919	-0.15	0.39	0.075	0.669	-0.007	0.967	0.035	0.84	-0.022	0.902	-0.424	0.055	0.211	0.359	0.083	0.72	0.252	0.27
R-ACR	0.312	0.068	-0.400*	0.017	-0.380*	0.024	-0.400*	0.017	0.264	0.126	-0.332	0.052	-0.313	0.067	-0.333	0.051	-0.248	0.279	0.14	0.546	0.069	0.766	0.17	0.46
L-ACR	0.211	0.224	-0.354*	0.037	-0.386*	0.022	-0.33	0.053	0.137	0.432	-0.232	0.18	-0.244	0.157	-0.221	0.203	-0.262	0.252	0.429	0.052	0.463*	0.035	0.402	0.071
R-SCR	-0.062	0.724	-0.239	0.167	-0.317	0.064	-0.18	0.3	0.027	0.879	-0.232	0.18	-0.265	0.124	-0.199	0.251	-0.045	0.846	0.18	0.434	0.211	0.359	0.155	0.503
L-SCR	0.066	0.708	-0.446**	0.007	-0.525**	0.001	-0.368*	0.03	0.092	0.598	-0.401*	0.017	-0.447**	0.007	-0.345*	0.042	-0.095	0.683	0.166	0.471	0.149	0.519	0.165	0.474
R-PCR	0.24	0.165	-0.343*	0.044	-0.360*	0.034	-0.326	0.056	0.172	0.323	-0.23	0.184	-0.233	0.178	-0.223	0.199	0.106	0.648	-0.213	0.353	-0.275	0.227	-0.174	0.45
L-PCR	0.232	0.18	-0.306	0.074	-0.279	0.104	-0.312	0.068	0.183	0.292	-0.23	0.183	-0.196	0.259	-0.243	0.159	-0.025	0.915	-0.161	0.486	-0.264	0.248	-0.099	0.67
R-PTR	0.267	0.121	-0.149	0.393	0.081	0.642	-0.235	0.175	0.135	0.438	-0.027	0.876	0.129	0.459	-0.096	0.584	0.108	0.641	-0.224	0.329	-0.329	0.145	-0.17	0.461
L-PTR	0.348*	0.04	-0.159	0.36	0.099	0.571	-0.27	0.117	0.201	0.247	-0.052	0.765	0.128	0.464	-0.137	0.433	0.152	0.512	-0.357	0.112	-0.503*	0.02	-0.254	0.266
R-SS	0.303	0.077	-0.31	0.07	-0.149	0.394	-0.336*	0.048	0.211	0.223	-0.159	0.361	-0.003	0.988	-0.202	0.246	-0.018	0.938	-0.234	0.308	-0.497*	0.022	-0.095	0.682
L-SS	0.27	0.117	-0.298	0.083	-0.208	0.231	-0.312	0.068	0.118	0.501	-0.145	0.405	-0.105	0.549	-0.151	0.385	0.134	0.562	-0.258	0.258	-0.361	0.108	-0.185	0.421
R-SFOF	-0.1	0.567	-0.08	0.648	-0.192	0.268	-0.016	0.927	-0.029	0.869	-0.144	0.409	-0.225	0.193	-0.094	0.592	-0.176	0.446	0.056	0.81	-0.006	0.98	0.082	0.724
L-SFOF	-0.118	0.5	-0.247	0.153	-0.396*	0.019	-0.12	0.494	0.013	0.94	-0.21	0.227	-0.255	0.139	-0.151	0.385	-0.361	0.108	0.118	0.61	-0.101	0.663	0.215	0.349

R-TAP	0.055	0.755	-0.124	0.479	-0.125	0.474	-0.118	0.498	-0.073	0.676	0.001	0.996	-0.019	0.915	0.011	0.948	0.265	0.245	-0.277	0.224	-0.286	0.209	-0.264	0.247
L-TAP	0.138	0.429	-0.281	0.102	-0.308	0.072	-0.253	0.142	0.124	0.478	-0.206	0.235	-0.223	0.199	-0.188	0.28	0.018	0.938	-0.128	0.581	-0.176	0.445	-0.093	0.689
